# Cyclic Guanosine Monophosphate-Dependent Protein Kinase I Stimulators and Activators Are Therapeutic Alternatives for Sickle Cell Disease

**DOI:** 10.4274/tjh.2017.0407

**Published:** 2018-03-06

**Authors:** Mohankrishna Ghanta, Elango Panchanathan, Bhaskar VKS Lakkakula

**Affiliations:** 1 Sri Ramachandra Medical College and Research Institute-DU, Faculty of Medicine, Department of Pharmacology, Chennai, Tamil Nadu, India; 2 Sickle Cell Institute Chhattisgarh, Department of Molecular Genetics, Division of Research, Raipur, Chhattisgarh, India

**Keywords:** Sickle cell anemia, cGK activation, Nitric oxide

## To the Editor,

Sickle cell anemia (SCA) can lead to a host of complications, including hemolysis, vaso-occlusive episodes (painful crises), pulmonary hypertension, acute chest syndrome, and multiorgan damage. SCA has no widely available cure. Furthermore, the available treatments have unfavorable side effects, such as myelosuppression of blood cells from continuous use of hydroxyurea, iron overload from repeated blood transfusions, or immunosuppressive treatments required to sustain a bone marrow transplant. In patients with SCA, hemoglobin-induced damage of endothelial cells can lead to endothelial dysfunction due to the deficiency of nitric oxide (NO) [1]. NO is continuously synthesized by the endothelium to maintain vascular tone. The NO-soluble guanylate cyclase (sGC)-cyclic guanosine monophosphate (cGMP) signaling (NO-sGC-cGMaP) pathway is one of the three important signaling pathways that are regulated by NO in maintaining the vascular tone. NO stimulates sGC in the vascular smooth muscle cells to induce formation of cGMP. This produced cGMP causes stimulation of cGMP-dependent protein kinases (cGKs), which in turn stimulate voltage-dependent ion channels. The cGKs are serene and threonine kinases, substrates for cGMP to elicit physiological functions. cGKs inhibit calcium release from the endoplasmic reticulum through the inositol 1,4,5-trisphosphate receptor-associated cGMP kinase substrate (IRAG) and alternatively activate myosin-light-chain phosphatase by inhibiting the MLC kinases, with both mechanisms resulting in smooth muscle relaxation [2]. Two types of cGKs have been revealed to date. Mammalian cGKs exist as two isoforms, cGKI and cGKII, which are coded by the *prkg1 *and *prkg2* genes, respectively. In humans two isoforms of cGKI have been described, cGKI-a and cGKI-b, differing only in their N-terminal parts and generated by alternative splicing of a single gene. Northern blot analysis revealed that human cGKI-a mRNA was present in the aorta, heart, kidneys, and adrenal glands. In contrast, human cGKI-b mRNA was present only in the uterus.

**Figure 1:**
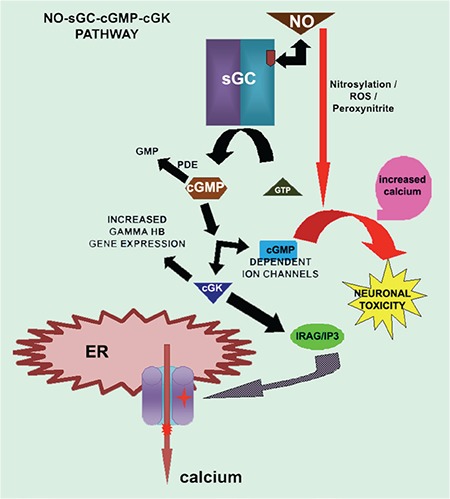
Schema of the nitric oxide-soluble guanylate cyclase-cyclic guanosine monophosphate-protein kinases I signaling pathway in the treatment of sickle cell anemia vasculopathies. *NO: Nitric oxide, sGC: soluble guanylate cyclase, cGMP: cyclic guanosine monophosphate, cGKI: protein kinases.*

In SCA, vascular tone control is compromised due to vasculopathy associated with hemolysis. As NO is considered beneficial, hydroxyurea and inhalational NO were administered to increase the bioavailability of NO, which raises cGMP levels [3]. Phosphodiesterases (PDEs) are enzymes that catalyze cGMP to GMP. Inhibitors of PDEs also increase cGMP levels by decreasing the degradation of cGMP. Inhibition of PDE9A enzyme with the specific inhibitor BAY73-6691 reversed the increased adhesive properties of neutrophils in sickle cell disease and increased production of the g-globin gene (*HBG*) in K562 cells. Furthermore, sGC activators were suggested for treatment of sickle cell disease (Figure 1) [4]. 

NO can lead to excess production of reactive oxygen species (ROS) and peroxynitrites. NO was also shown to induce cyclooxygenase and its isoforms, resulting in formation of prostaglandins, which leads to neuroinflammation [5]. NO also increases cGMP levels and leads to glutamate-induced toxicity resulting in neurodegeneration in the central nervous system (CNS) [6]. Furthermore, NO-dependent and NO-independent activators of sGC and inhibitors of PDEs tend to increase cGMP levels and similarly lead to glutamate toxicity and neurodegeneration in the CNS upon prolonged usage. The above-mentioned limitations show that there is a need for developing a potent drug similar to it with a safer pharmacological profile using the candidates of the pathway. Hence, another member of the same pathway, cGKI, can help as a therapeutic target, because cGK activity was reported to be spared on cGMP-dependent ion channels, which were shown to cause neurotoxicity [7]. cGKI activators that regulate IP3/IRAG calcium channels of the endoplasmic reticulum are therapeutically valuable and may change the phase of treatment. cGKI-b was reported to be abundant in platelets and inhibited platelet aggregation by decreasing intracellular calcium by blocking IRAG/IP3 calcium channels [8]. A study reported cGK’s regulatory role in stimulation of g-gene expression of fetal hemoglobin [9]. Activators of cGKI may provide drugs with safer pharmacological profiles in the treatment of SCA vasculopathies and pulmonary hypertension. To date, S-tides have been reported as activator drugs produced as synthetic peptides stimulating cGKI-a [10]. New drug discoveries targeting cGKI-a and cGKI-b may ensure safer pharmacological drug profiles of the NO-sGC-cGMP-cGK pathway in the treatment of SCA.
